# Neuromuscular characteristics of dynamic balance control in patients with sarcopenia: a combined analysis of surface electromyography and limits of stability

**DOI:** 10.3389/fnagi.2026.1835815

**Published:** 2026-07-08

**Authors:** Jingchun Wang, Ting Zhang, Qian Qian, Wenpeng Shan, Jie Xiang

**Affiliations:** 1 School of Medical Technology, Xuzhou Medical University, Xuzhou, China; 2 Wuxi Ninth People’s Hospital, Wuxi, China; 3 National Centre for Sport and Exercise Medicine, School of Sport, Exercise and Health Sciences, Loughborough University, Loughborough, United Kingdom; 4 Department of Rehabilitation Medicine, The Affiliated Hospital of Xuzhou Medical University, Xuzhou, China

**Keywords:** bilateral coordination, force platform, neuromuscular characteristics, sarcopenia, surface electromyography

## Abstract

**Introduction:**

Sarcopenia is a major risk factor for falls in older adults; however, the neuromuscular characteristics underlying dynamic balance deficits remain poorly understood.

**Objective:**

This study aimed to investigate balance performance and relative muscle contribution patterns in older adults with sarcopenia during dynamic limits of stability (LOS) tasks.

**Methods:**

Twenty-seven right-dominant older adults with sarcopenia and 27 age-matched non-sarcopenic controls were recruited. LOS tasks were performed using a force platform, while surface electromyography (sEMG) was recorded bilaterally from 10 lower-limb muscles. Amplitude contribution ratio (ACR), load bias ratio (LR), asymmetry index (AI), and Shannon entropy were analyzed.

**Results:**

The sarcopenia group exhibited reduced LOS performance across all directions, with greater impairment in the backward direction. LR-left was lower, whereas forward and backward AI values were higher in the sarcopenia group. Repeated-measures analyses revealed significant group effects for LR and AI, without significant direction-related interactions. ACR analysis showed a significant interaction among direction, muscle region, and group. Post hoc analyses indicated increased proximal and decreased distal relative muscle contributions only during the forward task. Shannon entropy was lower in the sarcopenia group.

**Conclusion:**

Older adults with sarcopenia exhibited direction-dependent impairments in dynamic balance performance, accompanied by altered proximal-to-distal relative muscle contribution patterns, asymmetric bilateral contribution distribution, and reduced diversity of muscle recruitment patterns. LR, AI, and Shannon entropy may serve as exploratory indices for characterizing relative muscle coordination during dynamic balance tasks and may provide additional insights for the assessment and rehabilitation of sarcopenia-related balance impairments.

## 1 Introduction

As the global population ages at an unprecedented rate, 28–35% of older adults experience falls annually (Meekes and Stanmore, 2017), often severely compromising quality of life and increasing healthcare and societal burden (Krauss et al., 2007; Haines et al., 2013; Jefferis et al., 2014). Sarcopenia, characterized by the progressive loss of skeletal muscle mass and functional capacity with age, is a well-recognized intrinsic risk factor for falls in geriatric populations (Choi and Jun, 2024). A key pathway linking sarcopenia to increased fall risk is impaired neuromuscular control, which compromises dynamic balance. Accordingly, dynamic balance performance represents an important functional marker of sarcopenia.

In recent years, a growing body of evidence has indicated that neuromuscular damage, rather than a simple loss of muscle mass, is a central driver of reduced mobility and functional decline in older adults. Age-related denervation, motor unit degradation, and structural and transmission defects at the neuromuscular junction can all lead to reduced motor unit activation rates and declines in muscle strength (Clark, 2019). Neurophysiological studies have further confirmed that the loss of central motor neurons, reduced firing rates, and altered intermuscular coordination disrupt the temporal patterns of motor output and muscle recruitment strategies, thereby impairing functional performance (Nùñez-Lisboa et al., 2023). These neurogenic alterations may occur in the early stages of sarcopenia and persist throughout the disease continuum.

Advances in diagnostic technologies have enhanced the identification and classification of sarcopenia. Dual-energy X-ray absorptiometry (DXA) is widely regarded as the gold standard for measuring muscle mass, while bioelectrical impedance analysis (BIA), MRI, and CT scans offer additional diagnostic insights (Cruz-Jentoft et al., 2019). However, these assessments have limitations: DXA exposes patients to ionizing radiation, both DXA and MRI are relatively expensive, require trained personnel, and are not readily available in routine clinical settings. Traditional balance assessments in older adults, such as single-leg stance, the Berg Balance Scale, and the Timed Up and Go test, are confined to static postures or simple dynamic tasks and thus fail to capture the multidimensional demands of postural control in daily life (Jonsson et al., 2004; Downs et al., 2013; Bohannon, n.d.). In contrast, surface electromyography (sEMG) is a non-invasive, low-cost technique that records the electrical activity of muscles, providing real-time information on muscle recruitment, coordination, and neuromuscular adaptation. The limits of stability (LOS) test quantifies the maximum displacement of the center of pressure in multiple directions, offering a more comprehensive and objective assessment of dynamic balance control (Shih et al., 2016). When combined with sEMG, which enables real-time characterization of muscle activation, LOS assessment not only provides performance-based outcomes but also offers insights into the neuromuscular responses underlying balance control.

In recent years, sEMG has been widely used to investigate neuromuscular alterations associated with sarcopenia. Multiple studies have examined lower limb muscles involved in maintaining daily activities and postural stability using EMG (Leone et al., 2022, 2025; Hirono et al., 2024; Kumar et al., 2024). EMG recordings acquired during isometric contractions and dynamic tasks (e.g., walking and sit-to-stand transitions) have revealed that individuals with sarcopenia often exhibit reduced muscle activation levels and altered EMG spectral characteristics (Ma et al., 2023; Sung et al., 2023; He et al., 2024). However, existing studies have largely focused on classifying EMG features using machine learning approaches, with less emphasis on their physiological significance and the underlying relationship with functional impairment (Leone et al., 2022; Junquera-Godoy et al., 2023; Ma et al., 2023; Kumar et al., 2024; Li et al., 2024). Furthermore, there is a paucity of research examining neuromuscular characteristics directly associated with fall risk using dynamic balance perturbation tasks, which has limited the understanding of early neuromuscular decline processes.

## 2 Materials and methods

### 2.1 Participants

A cohort of individuals diagnosed with sarcopenia was recruited from the geriatrics department of the hospital, while age-matched controls without sarcopenia were recruited from the community. The final sample comprised 27 participants in each group. Inclusion criteria included: (1) aged 60 years or older, (2) independent performance of basic activities of daily living without assistive devices, (3) absence of neurological, vestibular, or orthopedic conditions or injuries that could compromise balance, (4) no cognitive or affective disorders, (5) no visual deficits, (6) right leg dominance, and (7) no alterations in physical status or activity level on the day preceding assessment. Exclusion criteria were: (1) presence of neurological, vestibular, or orthopedic disorders or injuries affecting balance, (2) significant cognitive or emotional disturbances, (3) changes in physical status or activity level on the day before testing, and (4) incomplete assessments or missing data. Leg dominance was established using the kicking test, which evaluates lower limb strength and coordination; the limb achieving the greatest soccer ball displacement was identified as dominant (Hsu et al., 2022).

Sarcopenia was diagnosed in this study according to the AWGS 2019 guidelines (Chen et al., 2020). Muscle mass was assessed using a multi-frequency bioelectrical impedance body composition analyzer (InBody270, Biospace Co., Korea) by measuring the appendicular skeletal muscle mass index (ASMI). Low muscle mass was defined as ASMI < 7.0 kg/m^2^ in men or < 5.7 kg/m^2^ in women. Muscle strength was measured by dominant handgrip strength using a handgrip dynamometer (EH101, Senssun, China), with two trials performed and the maximum value recorded. Low muscle strength was defined as handgrip strength < 28 kg in men or < 18 kg in women. Physical performance was evaluated using a 6 m usual gait speed test, with two trials performed and the mean value recorded. Poor physical performance was defined as a gait speed < 1.0 m/s. According to the AWGS 2019 diagnostic criteria, possible sarcopenia was defined as low muscle strength or poor physical performance. Confirmed sarcopenia was defined as low muscle mass accompanied by low muscle strength and/or poor physical performance. Severe sarcopenia was defined by the coexistence of low muscle mass, low muscle strength, and poor physical performance. This study included only participants who met the criteria for confirmed sarcopenia and did not include those who met only the criteria for possible sarcopenia.

Based on prior literature (Kurz et al., 2012), an a priori power analysis conducted with G*Power version 3.1 indicated that a minimum of 21 participants per group was necessary to achieve a statistical power of 0.80 at an alpha level of 0.05. To account for an anticipated 20% dropout rate, approximately 27 participants were enrolled in each group to ensure sufficient statistical power. This study was approved by the Institutional Review Board of the corresponding author’s affiliated university (No. XYFY2021-KL269-01), and all participants provided written informed consent in accordance with the Declaration of Helsinki.

### 2.2 Experimental procedure

Participants first completed demographic data collection and the ASMI evaluation. They then performed the LOS assessment on a force platform integrated with a balance system, while sEMG signals were recorded simultaneously. All procedures were supervised by a certified therapist who was blinded to group allocation.

**FIGURE 1:**
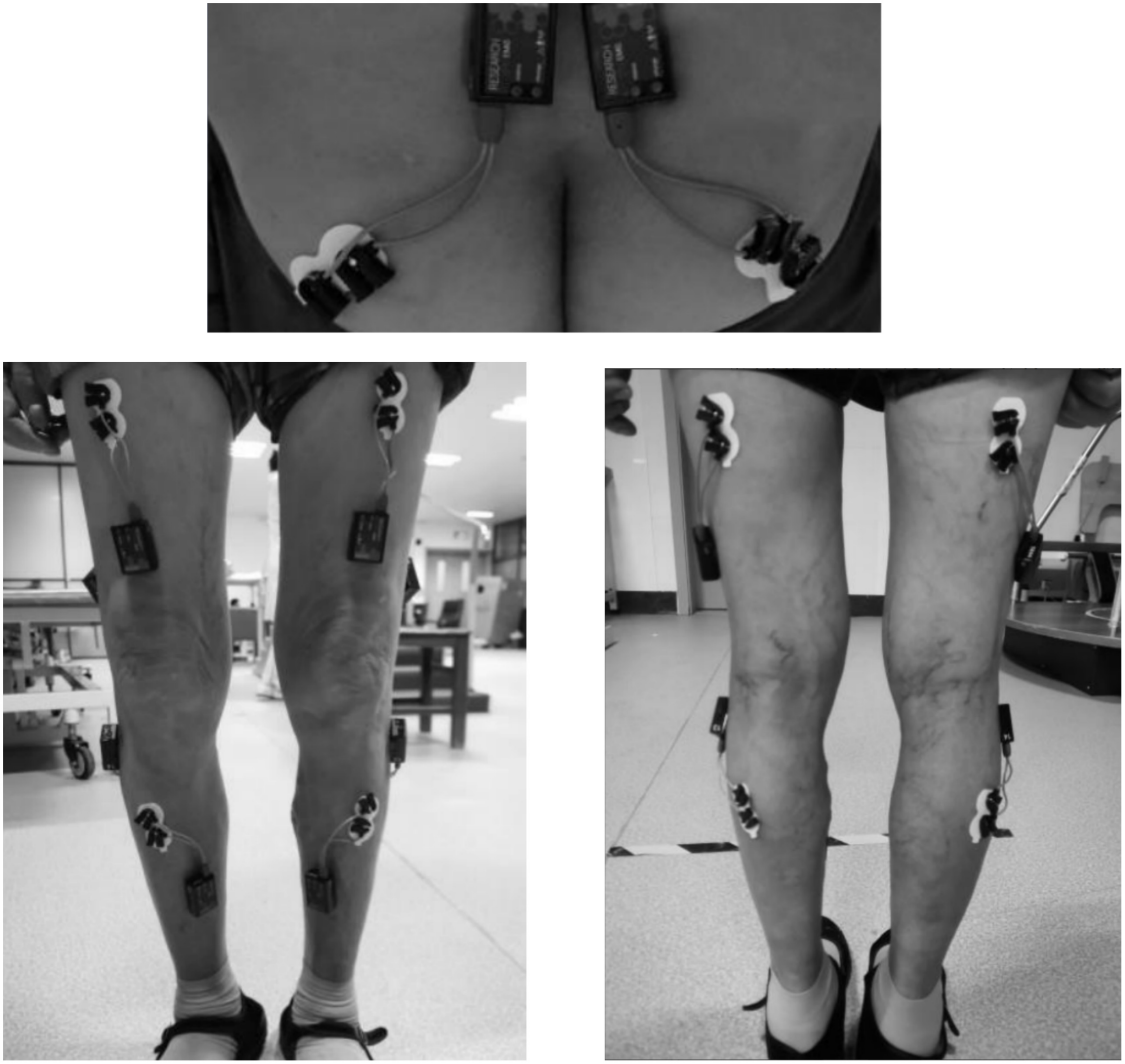
Schematic diagram of 10 surface electromyography muscle test points.

sEMG signals were acquired using a wireless Desktop DTS system (Noraxon Inc., Scottsdale, AZ, United States) featuring 16-bit resolution, a 2,000 Hz sampling rate, a common mode rejection ratio of 100 dB, and baseline noise (root mean square) below 1 μV. Electromyographic signals were recorded from 10 muscles, bilateral gluteus maximus (GM), rectus femoris (RF), long head of biceps femoris (LBF), tibialis anterior (TA), and lateral gastrocnemius (LGN) (Figure 1). These muscles were chosen for their anatomical relevance to the hip, knee, and ankle joints and their complementary roles as agonist-antagonist pairs, allowing comprehensive assessment of neuromuscular activation during LOS tasks.

Before electrode placement, skin preparation (shaving, abrasion, alcohol cleansing, air drying) minimized impedance. Wireless sEMG sensors were positioned near tendinous or bony landmarks, with disposable Ag/AgCl gel bipolar electrodes placed along muscle belly midlines, parallel to fibers, following European guidelines (Hsu et al., 2022). The desktop receiver connected to a computer via USB, with sEMG recording start/end points manually annotated for synchronization with the balance system.

**FIGURE 2:**
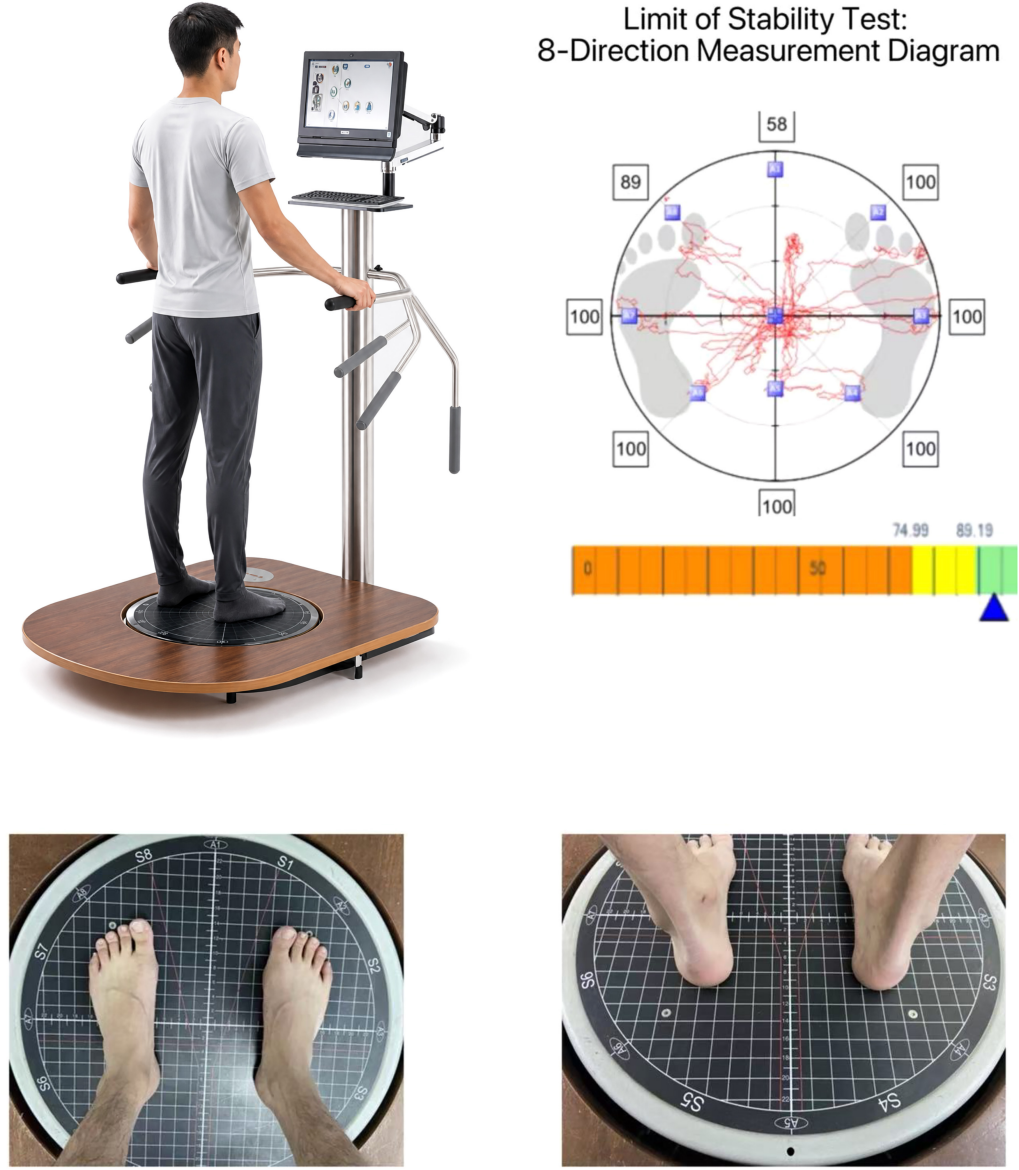
Schematic diagram of limits of stability measurement using a ProKin Line 252 force platform.

The ProKin Line 252 force platform (TecnoBody) used four transducers to monitor center of pressure (CoP) and postural sway (0.2° angular resolution, 20 Hz sampling). During LOS assessments, participants stood barefoot (in socks) with standardized foot placement for symmetrical weight distribution, arms relaxed, trunk upright, and gaze forward. Software displayed eight directional targets (anterior, anterior-right, right, posterior-right, posterior, posterior-left, left, anterior-left). Participants shifted CoP to each target within a time window, maintaining balance without heel lift, then returned to neutral; the sequence repeated for all directions (Figure 2).

Upon completion of the limits of stability test, participants proceeded to the second assessment task: maintaining a standing posture under unstable conditions. The force platform was configured to a free-swinging mode, and participants positioned both feet at the center of the platform according to the standard placement. Participants were instructed to maintain their body as stable as possible for 30 s, with arms hanging naturally at their sides, and to refrain from arm swinging or trunk compensatory movements. During the test, sEMG signals were simultaneously recorded from 10 bilateral lower limb muscles to assess the distribution characteristics and coordinative complexity of muscle activation patterns under sustained postural perturbation conditions.

### 2.3 Outcome assessment

#### 2.3.1 Limits of stability evaluation

Participants completed the LOS assessment utilizing synchronized sEMG and force platform data acquisition systems. Each trial followed a standardized protocol to ensure methodological consistency. Data are reported as mean ± standard deviation (SD). The normative LOS range is 75–100%, with 100% indicating successful contact with the blue target zone. Scores within this range were considered physiologically normal. Lower LOS values indicate impaired postural stability, and the lowest direction score identifies the participant’s greatest fall risk.

#### 2.3.2 Surface electromyography signal analysis

sEMG data were acquired, processed, and exported using MyoResearch XP software (version 3.16.96; Noraxon Inc., United States). Raw sEMG signals were filtered using a finite impulse response (FIR) band-pass filter with a frequency range of 10–500 Hz. The signals were subsequently full-wave rectified and smoothed using a root mean square (RMS) algorithm with a 150 ms moving window.

During the LOS test, sEMG and force platform data were recorded simultaneously. The start and end points of sEMG recording were marked in MyoResearch XP and aligned with the task periods defined by the balance testing system. The LOS test consisted of eight directional tasks, of which four primary directions—forward, backward, leftward, and rightward—were selected for sEMG contribution analysis. sEMG data were segmented according to the directional task periods defined by the balance system, and iEMG was calculated separately over the complete task duration for each direction. For the unstable platform standing task, the analysis window was defined as the entire 30 s testing period. Each task was repeated three times, and the mean value of valid trials was used for subsequent analyses.

All sEMG signals were visually inspected prior to analysis to ensure signal quality. Trials with electrode detachment, signal loss, signal saturation, abnormal noise, or obvious motion artifacts were excluded from analysis. No trials met the exclusion criteria in this study; therefore, all validly recorded trials were included in the subsequent iEMG and Amplitude contribution ratio (ACR) analyses.

Integrated electromyography (iEMG; μV⋅s) was calculated within the corresponding analysis windows using the standard EMG analysis module of MyoResearch XP. ACR was subsequently calculated based on iEMG values to characterize the relative contribution distribution among different muscle channels within the same task. The calculation formula was as follows: ACR (%) = (iEMG of a specific channel/sum of iEMG values across all channels) × 100 (Kurz et al., 2012). The ACR was calculated for the 10 lower-limb muscles. Based on the ACR, the load bias ratio, asymmetry index, and Shannon entropy were subsequently calculated.

Based on the methodology used for calculating the muscle utilization ratio (McDonald et al., 2018), the load bias ratio (LR) was defined as the percentage of total ACR on one side relative to the total contribution of both sides, and was used to quantify load distribution between the bilateral lower limb muscles during the limits of stability task. For the leftward and rightward limits of stability tasks, the load bias ratio was calculated as follows:


L⁢e⁢f⁢t⁢w⁢a⁢r⁢d⁢l⁢o⁢a⁢d⁢b⁢i⁢a⁢s⁢r⁢a⁢t⁢i⁢o=L⁢e⁢f⁢t⁢t⁢o⁢t⁢a⁢l⁢a⁢m⁢p⁢l⁢i⁢t⁢u⁢d⁢e⁢c⁢o⁢n⁢t⁢r⁢i⁢b⁢u⁢t⁢i⁢o⁢n (1)

r⁢a⁢t⁢i⁢o/(L⁢e⁢f⁢t+R⁢i⁢g⁢h⁢t⁢t⁢o⁢t⁢a⁢l⁢a⁢m⁢p⁢l⁢i⁢t⁢u⁢d⁢e⁢c⁢o⁢n⁢t⁢r⁢i⁢b⁢u⁢t⁢i⁢o⁢n⁢r⁢a⁢t⁢i⁢o)

    ×100%

R⁢i⁢g⁢h⁢t⁢w⁢a⁢r⁢d⁢l⁢o⁢a⁢d⁢b⁢i⁢a⁢s⁢r⁢a⁢t⁢i⁢o=R⁢i⁢g⁢h⁢t⁢t⁢o⁢t⁢a⁢l⁢a⁢m⁢p⁢l⁢i⁢t⁢u⁢d⁢e⁢c⁢o⁢n⁢t⁢r⁢i⁢b⁢u⁢t⁢i⁢o⁢n (2)

 ratio/(Left+Righttotalamplitudecontribution

    ratio)×100%.

A lower load bias ratio indicates reduced lateralization.

For the forward and backward limits of stability tasks, the asymmetry index (AI) was calculated as follows (Chen et al., 2023):


Forwardasymmetryindex=|Lefttotal (3)

  a⁢m⁢p⁢l⁢i⁢t⁢u⁢d⁢e⁢c⁢o⁢n⁢t⁢r⁢i⁢b⁢u⁢t⁢i⁢o⁢n⁢r⁢a⁢t⁢i⁢o-R⁢i⁢g⁢h⁢t⁢t⁢o⁢t⁢a⁢l⁢a⁢m⁢p⁢l⁢i⁢t⁢u⁢d⁢e

  contributionratio|

Backwardasymmetryindex=|Lefttotalamplitude (4)

c⁢o⁢n⁢t⁢r⁢i⁢b⁢u⁢t⁢i⁢o⁢n⁢r⁢a⁢t⁢i⁢o-R⁢i⁢g⁢h⁢t⁢t⁢o⁢t⁢a⁢l⁢a⁢m⁢p⁢l⁢i⁢t⁢u⁢d⁢e

  contributionratio|

A larger index indicates a more pronounced difference in activation between the left and right sides.

For the unstable platform standing task, Shannon entropy was calculated using the ACR of the lower limb muscles to assess the complexity of muscle activation patterns (Hong et al., 2026).


$$H=-\sum_{i=1}^{10}\left(p_{i}\,\log_{2}\,\left(p_{i}\right)\right)$$ (5)

Before calculating Shannon entropy, the ACR values of the 10 muscles were first normalized into probability values with a total sum of 1. Entropy was subsequently calculated using a base-2 logarithm. In this equation, p_*i*_ represents the normalized ACR value of the i-th muscle, and *n* = 10 represents the number of muscle channels included in the analysis. A higher entropy value indicates a more dispersed distribution of relative muscle contributions, whereas a lower entropy value indicates a more concentrated distribution.

### 2.4 IBM SPSS statistics

Statistical analyses were performed using IBM SPSS Statistics 27.0 (IBM Corp., Armonk, NY, United States). The normality of continuous variables was first assessed using the Shapiro–Wilk test. Normally distributed data are presented as mean ± standard deviation, and between-group comparisons were conducted using independent-samples *t*-tests. Non-normally distributed data are presented as median (interquartile range), and between-group comparisons were performed using the Mann–Whitney U test. Categorical variables were compared using the chi-square test or Fisher’s exact test.

LOS data were analyzed using two-way repeated-measures ANOVA, with group (control vs. sarcopenia) as the between-subject factor and direction as the within-subject factor. ACR data were analyzed using three-way repeated-measures ANOVA with group, direction, and muscle region (proximal vs. distal) as factors. Bonferroni correction was applied for *post-hoc* pairwise comparisons. LR and AI variables were compared between groups using the Mann–Whitney U test and further analyzed using two-way mixed-design repeated-measures ANOVA to examine the effects of group, direction, and their interaction. Bonferroni correction was applied for *post-hoc* pairwise comparisons. Shannon entropy was compared between groups using the Mann–Whitney U test. All tests were two-tailed, with statistical significance set at *p* < 0.05.

## 3 Results

### 3.1 Baseline characteristics

No significant differences were observed in age or body mass index between the sarcopenia and control groups (all *P* > 0.05). These results were consistent across both male and female subgroups (Table 1), and there was no statistically significant difference in sex distribution between the two groups (χ^2^ = 0.307, *P* = 0.580). In addition, compared with the control group, both male and female participants in the sarcopenia group showed significantly lower ASMI, handgrip strength, and 6 m gait speed (all *P* < 0.001). Among the 54 participants, 27 (50.0%) were classified as non-sarcopenic, 10 (18.5%) as sarcopenic, and 17 (31.5%) as severely sarcopenic.

**TABLE 1:** Baseline characteristics and sarcopenia diagnostic indicators of participants.

Variables	Control group (*n* = 27)	Sarcopenia group (*n* = 27)	*P*-value
Baseline characteristics			
Age (years)	71.85 ± 4.24 (65–82)	74.56 ± 6.89 (60–88)	0.088
Sex, n (%)			0.58
Male	10 (37.0)	12 (44.4)	
Female	17 (63.0)	15 (55.6)	
BMI (kg/m^2^)	21.95 ± 2.56	20.89 ± 2.37	0.12
Sarcopenia Diagnostic indicators			
ASMI (kg/m2)			
Male	7.41 ± 0.70	6.16 ± 0.41	< 0.001
Female	6.15 ± 0.58	5.31 ± 0.28	< 0.001
Handgrip strength (kg)			
Male	34.12 ± 4.23	22.64 ± 3.85	< 0.001
Female	24.30 ± 6.55	16.80 ± 3.90	< 0.001
Gait speed (m/s)			
Male	1.09 ± 0.11	0.89 ± 0.10	< 0.001
Female	1.14 ± 0.12	0.87 ± 0.11	< 0.001

Data are presented as mean ± standard deviation or n (%). BMI, body mass index; ASMI, appendicular skeletal muscle mass index. *P*-values are provided for baseline comparability variables only, including age, sex distribution, and BMI. ASMI, handgrip strength, and gait speed are presented as diagnostic indicators to allow verification of sarcopenia classification according to the AWGS 2019 criteria. Low muscle mass was defined as ASMI < 7.0 kg/m^2^ in men and < 5.7 kg/m^2^ in women; low muscle strength as handgrip strength < 28 kg in men and < 18 kg in women; and reduced physical performance as usual gait speed < 1.0 m/s.

### 3.2 Comparative analysis of limits of stability

Two-way repeated-measures ANOVA (Table 2) showed significant main effects of group and direction (both *p* < 0.0001), as well as a significant Group × Direction interaction [*F*(7, 364) = 4.944, *p* < 0.001, partial η^2^ = 0.087], indicating direction-dependent differences in LOS impairment in individuals with sarcopenia. Bonferroni-corrected post hoc comparisons showed that the sarcopenia group had significantly lower LOS values in all eight directions and total LOS compared with the control group (all adjusted *p* < 0.0001). The largest between-group differences were observed in the posterior directions (backward, backward-left, and backward-right), with Cohen’s d values ranging from 2.25 to 2.92, indicating very large effect sizes. These findings suggest that individuals with sarcopenia exhibit overall reductions in limits of stability, with varying degrees of impairment across directions.

**TABLE 2:** Comparison of limits of stability in different directions and overall LOS between sarcopenia group and control group.

Direction	Control group mean	Sarcopenia group mean	Mean difference (95% CI)	Adjusted *P*-value	Cohen’s d
LOS1	81.34	56.32	25.01*** (18.07, 31.96)	< 0.001	1.97
LOS2	76.45	42.86	33.59*** (25.09, 42.09)	< 0.001	2.16
LOS3	70.04	36.54	33.49*** (28.20, 38.79)	< 0.001	3.46
LOS4	79.69	45.93	33.76*** (24.99, 42.53)	< 0.001	2.10
LOS5	78.17	59.97	18.20*** (9.96, 26.43)	< 0.001	1.21
LOS6	89.28	49.94	39.34*** (30.62, 48.06)	< 0.001	2.46
LOS7	84.00	45.43	38.57*** (31.35, 45.79)	< 0.001	2.92
LOS8	90.97	53.60	37.37*** (28.30, 46.44)	< 0.001	2.25
Overall LOS	74.65	44.83	29.83*** (20.91, 38.75)	< 0.001	1.83

The symbol *** indicates *p* < 0.001.

All comparisons were performed using two-way repeated-measures ANOVA followed by Bonferroni *post-hoc* tests. Adjusted *P*-values account for multiple comparisons across eight directions. LOS1: right lateral; LOS2: right-forward; LOS3: forward; LOS4: left-forward; LOS5: left lateral; LOS6: left-backward; LOS7: backward; LOS8: right-backward. Overall LOS represents the composite score across all directions. CI = confidence interval. * *p* < .05, ** *p* < 0.01, *** *p* < 0.001.

### 3.3 Bilateral coordination: load bias ratio and asymmetry index

**FIGURE 3:**
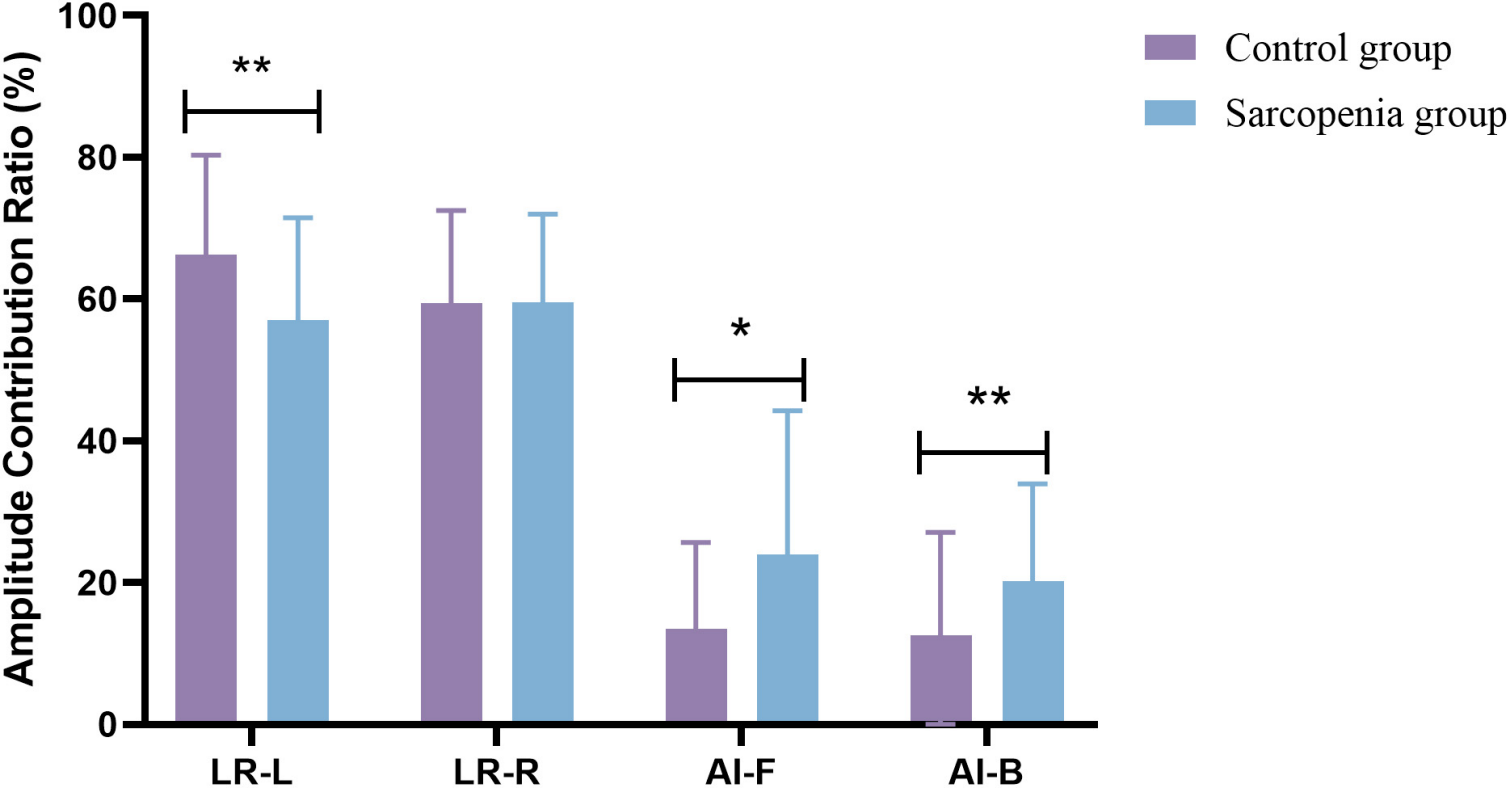
Comparison of load ratio, asymmetry index between groups. LR-L, leftward load ratio; LR-R, rightward load ratio; AI-F, forward asymmetry index; AI-B, backward asymmetry index. *P < 0.05, **P < 0.01.

By calculating the LR for the left-right LOS tasks and the AI for the forward-backward LOS tasks, bilateral coordinative control was further assessed in individuals with sarcopenia (Figure 3 and Table 3). The sarcopenia group showed a significantly lower LR-left than the control group (58.75% vs. 68.43%, *p* = 0.0029), whereas no significant between-group difference was observed for LR-right (*p* = 0.8069).

**TABLE 3:** Comparison of load ratio, asymmetry index, and Shannon entropy between groups.

Variable	Control group (*n* = 27)	Sarcopenia group (*n* = 27)	*Z*	*p*	Effect size	Bonferroni correction
LR-left	68.43(62.87–73.9)	58.75(53.21–64.30)	−2.93	0.0029	0.40	Significant*
LR-right	63.59(58.24–68.94)	59.47(54.12–64.82)	−0.25	0.8069	0.03	ns
AI forward	10.64(6.21–15.07)	19.13(12.45–25.81)	−2.50	0.0119	0.34	Significant*
AI backward	6.64(3.52–9.76)	19.75(13.68–26.82)	−2.59	0.0092	0.35	Significant*
Shannon entropy	3.020(2.89–3.15)	2.835(2.71–2.96)	−3.81	< 0.001	0.52	–

The symbol * indicates statistical significance after Bonferroni correction, with the adjusted alpha level set at *p* < 0.0125.

For AI, the sarcopenia group demonstrated significantly higher values than the control group in both the forward direction (19.13% vs. 10.64%, *p* = 0.0119) and backward direction (19.75% vs. 6.64%, *p* = 0.0092). Further repeated-measures ANOVA showed a significant main effect of group for AI [*F*(1, 52) = 8.657, *p* = 0.005, partial η^2^ = 0.143], whereas neither the main effect of direction [*F*(1,52) = 0.636, *p* = 0.429, partial η^2^ = 0.012] nor the Group × Direction interaction [*F*(1, 52) = 0.238, *p* = 0.627, partial η^2^ = 0.005] reached statistical significance. Similar results were observed for LR, with a significant main effect of group [*F*(1, 52) = 4.474, *p* = 0.039, partial η^2^ = 0.079], but non-significant main effects of direction [*F*(1, 52) = 0.521, *p* = 0.474] and Group × Direction interaction [*F*(1, 52) = 2.510, *p* = 0.119, partial η^2^ = 0.046] (Table 4).

**TABLE 4:** Repeated-measures ANOVA results for load bias ratio and asymmetry index during LOS tasks.

Variable	Effect	*F*(df1,df2)	*P*	Partial η^2^
AI	Group	8.657 (1,52)	0.005*	0.143
	Direction	0.636 (1,52)	0.429	0.012
	Group × direction	0.238 (1,52)	0.627	0.005
LR	Group	4.474 (1,52)	0.039*	0.079
	Direction	0.521 (1,52)	0.474	0.010
	Group × direction	2.510 (1,52)	0.119	0.046

AI, asymmetry index; LR, load bias ratio. *P < 0.05.

Data are presented as median (interquartile range, IQR). Between-group comparisons were performed using Mann-Whitney U tests. A Bonferroni correction was applied for multiple comparisons across four variables, with statistical significance set at an adjusted alpha level of *p < 0.0125 (0.05/4). Shannon entropy was analyzed as a single independent test and thus not corrected. LR-left, leftward load ratio; LR-right, rightward load ratio; AI-forward, forward asymmetry index; AI-backward, backward asymmetry index.

### 3.4 Direction-specific muscle recruitment patterns: Changes in proximal and distal relative contributions

Repeated-measures ANOVA (Table 5) revealed significant Direction × Muscle region [*F*(3, 156) = 15.015, *p* < 0.001, partial η^2^ = 0.224] and Group × Muscle region interactions [*F*(1, 52) = 6.208, *p* = 0.016, partial η^2^ = 0.107] for ACR. A significant Direction × Muscle region × Group interaction was also observed [*F*(3, 156) = 2.736, *p* = 0.046, partial η^2^ = 0.050], indicating distinct proximal-to-distal muscle activation patterns between groups across task directions. Bonferroni-corrected post hoc analyses further showed that the sarcopenia group exhibited significantly higher proximal ACR and lower distal ACR during the forward task (both adjusted *p* = 0.002, Cohen’s *d* = 1.06–1.07). In contrast, no significant between-group differences were observed in the backward or leftward directions after multiple-comparison correction. Although proximal ACR in the rightward direction showed a moderate effect size (*d* = 0.67), the difference did not remain significant after correction (*p* = 0.141).

**TABLE 5:** Bonferroni-adjusted *post-hoc* comparisons of proximal and distal muscle amplitude contribution ratios during limit of stability tasks in the sarcopenia and control groups.

Condition	Control group Mean	Sarcopenia group Mean	Mean difference (95% CI)	Adjusted *P*-value	Cohen’s *d*
Forward-proximal	49.35	63.06	−13.72** (−20.73, −6.70)	0.002	1.07
Forward-distal	50.60	36.94	13.67** (6.65, 20.68)	0.002	1.06
Backward-proximal	40.92	43.30	−2.39 (−10.44, 5.67)	1.000	0.16
Backward-distal	59.03	56.70	2.33 (−5.72, 10.39)	1.000	0.16
Left-proximal	47.37	51.47	−4.09 (−12.24, 4.05)	1.000	0.27
Left-distal	52.70	48.53	4.17 (−3.97, 12.31)	1.000	0.28
Right-proximal	46.48	55.48	−9.00 (−16.36, −1.64)	0.141	0.67
Right-distal	51.28	44.52	6.76 (−1.60, 15.13)	0.886	0.44

Proximal muscles include the gluteus maximus, biceps femoris, and rectus femoris; distal muscles include the tibialis anterior and gastrocnemius. *Post-hoc* comparisons were adjusted using the Bonferroni correction.

**p < 0.01.

### 3.5 Diversity of relative muscle contribution patterns during the unstable standing task: Shannon entropy

**FIGURE 4:**
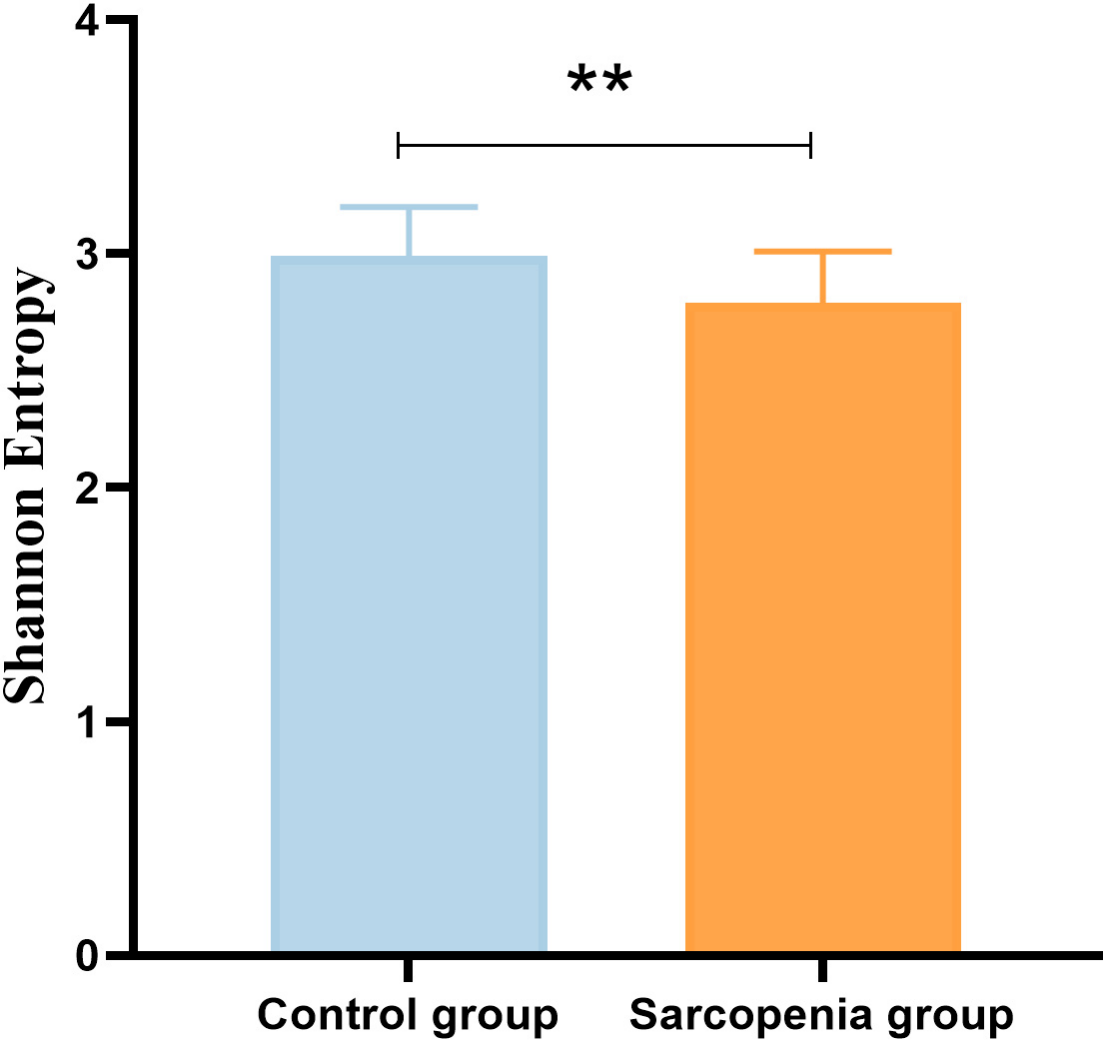
Comparison of Shannon entropy between groups during unstable platform testing. **P < 0.01, indicating significant differences between the sarcopenia group and control group.

During the unstable force platform balance task, we calculated Shannon entropy based on the ACR of the 10 lower limb muscles to assess neuromuscular coordination complexity. The sarcopenia group demonstrated a median Shannon entropy of 2.835 (IQR: 2.71–2.96), which was significantly lower than that of the control group (3.020, IQR: 2.89–3.15; Z = −3.81, *p* < 0.001, *r* = 0.52, indicating a large effect size) (Figure 4 and Table 3).

## 4 Discussion

This study simultaneously recorded surface electromyography signals and force platform limits-of-stability data to characterize balance performance, relative muscle contribution distribution, and coordination patterns in patients with sarcopenia during dynamic balance tasks. The results indicate that patients with sarcopenia exhibited diminished balance performance during the limits-of-stability task, along with direction-specific alterations including increased relative contributions of proximal muscle groups, asymmetric bilateral muscle contribution distribution, and reduced Shannon entropy. It should be noted that the ACR index employed in this study reflects the within-task relative contribution of each EMG channel’s integrated EMG (iEMG) to the total iEMG, and is therefore more appropriate for characterizing muscle sharing and contribution distribution patterns during dynamic balance tasks.

Our findings demonstrate that during the LOS test, the sarcopenia group exhibited significantly smaller LOS values than the control group across all eight directions (anterior, anterior-right, right, posterior-right, posterior, posterior-left, left, and anterior-left), consistent with the findings reported by Gadelha et al. (2018). A significant group × direction interaction effect was identified, suggesting that balance impairment in patients with sarcopenia does not manifest as a uniform decline across all directions, but rather exhibits pronounced directional heterogeneity. In particular, the most pronounced between-group differences were observed in the posterior directions (posterior, posterior-left, and posterior-right), indicating that stability during posterior weight-shifting tasks is disproportionately compromised in patients with sarcopenia. These findings are consistent with those of Yang et al. regarding fall patterns in older adults, thereby corroborating the validity of our observations (Yang et al., 2020). Our results indicate that poorer posterior LOS performance in sarcopenic patients may be associated with an elevated risk of backward falls, although the underlying mechanisms warrant further investigation.

This study conducted an exploratory analysis of bilateral muscle contribution distribution in older adults with sarcopenia during LOS tasks using LR and AI. The results showed that the leftward LR was significantly lower in the sarcopenia group than in the control group, suggesting a reduced relative contribution of the left-side muscles during the leftward leaning task. This finding may reflect altered bilateral muscle recruitment patterns during lateral balance tasks. However, no significant between-group difference was observed for the rightward LR. Further repeated-measures ANOVA revealed that the Group × Direction interaction for LR did not reach statistical significance, indicating that the overall pattern of between-group differences was generally similar across leftward and rightward directions. Although no significant directional interaction was observed, the between-group difference in leftward LR may still suggest altered lateral weight-shifting control strategies. This phenomenon may be related to the fact that all participants in the present study were right-leg dominant (Zhang et al., 2024). Because the right side was the dominant side, participants may have relied more on pre-existing dominant-side motor control strategies during rightward weight shifting, which may have partially attenuated the between-group differences and contributed to the relatively stable rightward LR findings (Sung and Lee, 2025).

In addition, the forward and backward AI values were higher in the sarcopenia group than in the control group, suggesting greater asymmetry in bilateral muscle contribution distribution during dynamic balance tasks. Further repeated-measures ANOVA showed that the Group × Direction interaction for AI did not reach statistical significance, indicating that the increased AI showed a generally similar pattern in the forward and backward tasks, without clear direction-specific differences.

Previous studies have suggested that age-related motor unit remodeling, muscle fiber denervation, and alterations in bilateral motor control may contribute to changes in muscle recruitment patterns observed in older adults. Nilwik et al. (2013) reported that type II muscle fibers are particularly susceptible to denervation during aging and may subsequently undergo reinnervation remodeling. Guo et al. (2024) reported reduced common synaptic input to motor units and diminished neuromuscular control capacity in older adults. Pratt et al. (2022) report demonstrated that handgrip strength asymmetry is associated with sarcopenia. These findings may provide potential explanations for the bilateral contribution distribution differences observed in the present study.

It should be noted that, because MVC normalization was not performed in this study, the present findings should be interpreted as changes in relative muscle contribution patterns within the task rather than as direct differences in absolute muscle activation levels or neural drive capacity. Therefore, the present results may reflect altered bilateral coordination strategies during dynamic balance tasks in older adults with sarcopenia compared with healthy older adults.

In the anterior LOS task, sarcopenic patients showed a direction-specific pattern of increased proximal and decreased distal muscle contributions. Compared with controls, the sarcopenia group showed significantly higher proximal and lower distal muscle contributions. This shift may relate to the frequency of anterior center-of-mass displacement in daily life, as forward leaning is the most common postural perturbation. Sarcopenic patients may adopt a proximally dominant muscle-sharing pattern during anterior dynamic balance control (Zhang et al., 2024; Cai et al., 2025). Cai et al. (2025) similarly found enhanced proximal and reduced distal muscle activation during walking in sarcopenic patients, proposing this as a neuromuscular adaptation specific to sarcopenia. However, whether this proximally dominant pattern improves balance or reduces fall risk was not verified. Its role as a true compensatory strategy remains unclear.

A similar proximal dominance was not observed in posterior or lateral directions, which may relate to the more pronounced posterior LOS deficits in sarcopenic patients. Lateral and posterior postural control involves more complex multi-joint coordination, trunk stabilization, and bilateral load regulation. Compensatory patterns may thus be more distributed, without the same degree of proximal dominance as in anterior tasks (Jasimi Zindashti et al., 2025).

From a neurophysiological perspective, the selective atrophy of fast-twitch muscle fibers—a core pathological feature of sarcopenia—has a greater impact on distal muscle groups. Distal muscles such as the tibialis anterior and gastrocnemius contain a higher proportion of fast-twitch fibers, which are essential for rapid postural adjustments (Reid et al., 2012; Sayed et al., 2016). Their atrophy may reduce distal muscle responsiveness. The increased proximal contribution may reflect task-related recruitment adjustments in older adults (Zhang et al., 2024; Cai et al., 2025). The proximal-to-distal shift in anterior tasks therefore likely reflects an altered muscle contribution ratio during dynamic balance control in sarcopenic patients. Whether this pattern carries functional compensatory significance warrants further study.

Sarcopenic patients showed significantly lower Shannon entropy than controls during the unstable platform balance task. This suggests a more concentrated distribution of relative lower-limb muscle contributions and reduced diversity in muscle recruitment patterns (Hong et al., 2026). It should be noted that Shannon entropy in this study primarily describes the within-task distribution of relative muscle contribution patterns across different muscle groups. Higher entropy indicates a more dispersed distribution of muscle contributions and greater recruitment diversity. Lower entropy suggests a more uniform recruitment pattern, potentially reflecting reduced postural regulation flexibility (Arjunan et al., 2013; Junquera-Godoy et al., 2025). Junquera-Godoy et al. (2025) similarly found reduced surface EMG complexity in sarcopenic patients during specific time windows following postural perturbation, indicating decreased variability in muscle recruitment patterns. Prior research suggests this change may be related to age-related motor unit loss, altered neuromuscular coordination, and reduced muscle functional reserve (Zhang et al., 2024; Junquera-Godoy et al., 2025). However, surface EMG signals are susceptible to subcutaneous tissue, muscle structure, and signal processing methods. The underlying neurophysiological mechanisms therefore cannot be directly verified in this study. Furthermore, the reduced Shannon entropy observed in this study may indicate that sarcopenic patients adopt a more stereotyped muscle recruitment pattern when facing unstable postural tasks. This may be associated with diminished adaptability to sudden postural perturbations and an elevated fall risk (Gadelha et al., 2018).

Sarcopenic patients may rely more heavily on proximal muscle recruitment during dynamic balance tasks, accompanied by asymmetric bilateral muscle contributions and reduced diversity in recruitment patterns. As the condition progresses, these altered recruitment patterns may be associated with multi-directional balance decline. Posterior stability appears more severely impaired, suggesting that posterior postural control may be a particular vulnerability in sarcopenic patients. The present findings primarily reflect changes in within-task relative muscle contribution distribution. They do not directly establish a clear functional compensatory role. These findings may nonetheless offer useful directions for future rehabilitation research. Insufficient activation of distal muscles, particularly the tibialis anterior, is a key contributor to balance impairment in sarcopenic patients. Rehabilitation should therefore emphasize ankle muscle strengthening. Cai et al. (2025) similarly suggest that sarcopenia rehabilitation should not focus solely on proximal large muscle groups, but should incorporate ankle muscle training into comprehensive intervention programs. Additionally, bilateral coordination training and posterior postural control training may warrant further attention (Kurz et al., 2016). To enhance clinical readability, we summarized the potential functional implications and possible rehabilitation directions corresponding to the main findings (Table 6).

**TABLE 6:** Summary of sarcopenia-related findings and potential clinical/rehabilitation implications.

Sarcopenia-related findings	Potential functional implication	Possible rehabilitation consideration
Reduced LOS across directions	Impaired dynamic balance control	Multi-direction balance training
Greater impairment in backward directions	Reduced posterior stability margin	Backward weight-shifting training
Increased AI in forward/backward directions	Altered bilateral coordination	Symmetry-oriented balance tasks
Direction-specific proximal shift in relative contribution	Task-dependent muscle recruitment redistribution	Hip–knee coordination exercises; Ankle dorsiflexor and plantarflexor strengthening.
Reduced Shannon entropy	Less diverse muscle contribution patterns	Variable and adaptive balance challenges

LOS, limits of stability; AI, asymmetry index.

This study has several limitations. MVC normalization was not applied, so between-subject amplitude comparisons should be interpreted with caution. The percentage contribution analysis describes within-task relative muscle contributions but cannot fully eliminate the influence of electrode placement, subcutaneous tissue, and signal crosstalk on sEMG interpretation. Muscle activation timing and co-activation temporal characteristics were not analyzed. As participants were community-dwelling older adults, findings may not generalize to frail or institutionalized populations. Future studies incorporating MVC normalization, high-density surface EMG, motor unit decomposition, and prospective follow-up are needed to further clarify neuromuscular recruitment characteristics and their clinical implications in sarcopenic patients.

## 5 Conclusion

Older adults with sarcopenia exhibited direction-dependent impairments in dynamic balance performance, accompanied by altered proximal-to-distal relative muscle contribution patterns, asymmetric bilateral contribution distribution, and reduced diversity of muscle recruitment patterns. LR, AI, and Shannon entropy may serve as exploratory indices for characterizing relative muscle coordination during dynamic balance tasks and may provide additional insights for the assessment and rehabilitation of sarcopenia-related balance impairments. However, the value of these indicators as fall risk predictors, clinical biomarkers, or rehabilitation targets requires further validation in prospective and interventional studies.

## Data Availability

The raw data supporting the conclusions of this article will be made available by the authors, without undue reservation.
